# Variability of Polychaete Secondary Production in Intertidal Creek Networks along a Stream-Order Gradient

**DOI:** 10.1371/journal.pone.0097287

**Published:** 2014-05-09

**Authors:** Tianjiang Chu, Qiang Sheng, Sikai Wang, Jihua Wu

**Affiliations:** Ministry of Education Key Laboratory for Biodiversity Science and Ecological Engineering, and Institute of Biodiversity Science, Fudan University, Shanghai, China; North Carolina State University, United States of America

## Abstract

Dendritic tidal creek networks are important habitats for sustaining biodiversity and ecosystem functioning in salt marsh wetlands. To evaluate the importance of creek heterogeneity in supporting benthic secondary production, we assess the spatial distribution and secondary production of a representative polychaete species (*Dentinephtys glabra*) in creek networks along a stream-order gradient in a Yangtze River estuarine marsh. Density, biomass, and secondary production of polychaetes were found to be highest in intermediate order creeks. In high order (3rd and 4th) creeks, the density and biomass of *D. glabra* were higher in creek edge sites than in creek bottom sites, whereas the reverse was true for low order (1st and 2nd) creeks. Secondary production was highest in 2nd order creeks (559.7 mg AFDM m^−2^ year^−1^) and was ca. 2 folds higher than in 1st and 4th order creeks. Top fitting AIC models indicated that the secondary production of *D. glabra* was mainly associated with geomorphological characters including cross-sectional area and bank slope. This suggests that hydrodynamic forces are essential factors influencing secondary production of macrobenthos in salt marshes. This study emphasizes the importance of microhabitat variability when evaluating secondary production and ecosystem functions.

## Introduction

The primary production in salt marshes can exceed 3900 g C m^−2^yr^−1^, making them among the most productive ecosystems in the world [1]. This high level of primary production forms the energy base for abundant animal consumers, including benthic macrofauna, nekton, and shorebirds, and thereby sustains a high level of secondary production [2], [3]. In the salt marshes of the northern Gulf of Mexico, secondary production exceeds other regions of the United States, which is exemplified by large fishery catches of penaeid shrimps (*Farfantepenaeus aztecus*, *F. duorarum* and *Litopenaeus setiferus* of 66% U.S.) and blue crabs (*Callinectes sapidus* of 25% U.S.) [4]. Similarly, researchers estimated that the secondary production of crabs (*Chiromantes dehaani*, *Helice tientsinensis*) in a Yangtze River estuarine salt marsh was 49.4 g AFDM m^−2^year^−1^, which is higher than that reported from other types of coastal wetlands at a similar latitude [5].

Secondary production is the formation of heterotrophic biomass through time [6]. It represents the cumulative responses of a population to abiotic and biotic stresses, and is an essential variable when quantifying food webs and ecosystem functioning such as material cycling and energy flow [7]. The habitat heterogeneity has been noted when estimating secondary productions. The secondary production of macrobenthos was highest in sandy organic sediment and lowest in muddy sediment in a southern New England salt marsh [8]. Secondary production of macrobenthos differed with vegetation types in the Mobile-Tensaw Delta [9]. A better understanding of the spatial differences in production, especially at local scales, would be helpful to assess the spatial variability of ecosystem functioning.

Intertidal creeks are an important sub-habitat in salt marsh ecosystems and are used by shorebirds and benthic, nektonic, and planktonic animals [10]. These habitats play a key role in promoting material cycle and energy flow between the salt marsh and offshore ecosystem [11]. According to the stream order classification, the smallest headwater stream is defined as a 1st order creek, and creek order increases thereafter when two creeks of the same order connect [12]. Geomorphological characters (e.g. length, width, and depth) and physicochemical properties (e.g. velocity, water temperature and substrate) are usually different between creeks of different orders [13], [14], [15]. Thus, creek order could be an important factor for evaluating the effects of local habitat heterogeneity on secondary production.

Polychaetes are the dominant macrobenthic component in many tidal creek ecosystems [16]. Their density can exceed 14,500 ind m^−2^ and serve as an important food source for higher trophic level consumers [17], [18]. They are also the prominent ecosystem engineers in salt marsh ecosystems. They can affect the organic matter content in sediment via bioturbation [19], accelerate the erosion of intertidal creeks [20], and stimulate the expansion of some plant species [21]. An understanding of the spatial variability in secondary production of this functionally important organism is essential for determining the processes that affect benthic ecosystems across different habitats. As to polychaete, the highest secondary production of *Caulleriella caputesocis* was found in the sediment containing 60 to 100% silt [22]. In the Río de la Plata estuary, secondary production of *Laeonereis culveri* was higher in creeks than in intertidal flat [17]. There are various microhabitats within tidal creek networks, but little is known about the spatial variability in polychaete production.

*Dentinephtys glabra* (Hartman), which belongs to the family Nephtyidae, is the most numerically dominant macrofaunal species in the creek systems of the Yangtze River estuarine marshes [23], [24]. *D. glabra* is a typical infaunal species which has low mobility and is considered an ideal and representative species to study the effects of sediment environmental variability [25]. This species is also a key link between primary producers and higher trophic levels such as nektonic fish and shorebirds, and thus plays an important role in the food web of the marsh ecosystem. To unravel the importance of creek heterogeneity on macrobenthos, we selected this representative polychaete species to evaluate the effects of creek order on benthic population and secondary production. We aim to answer the question how the distribution and secondary production of *D. glabra* differs among creeks of different orders.

## Material and Methods

### Ethics statement

This study was conducted according to the regulations of local and central government. Sample collections necessary to scientific research were authorized by the Specifications for oceanographic survey - Part 6: Marine biological survey (GB/T 1263.6 - 2007).

### Study site

**Figure 1:**
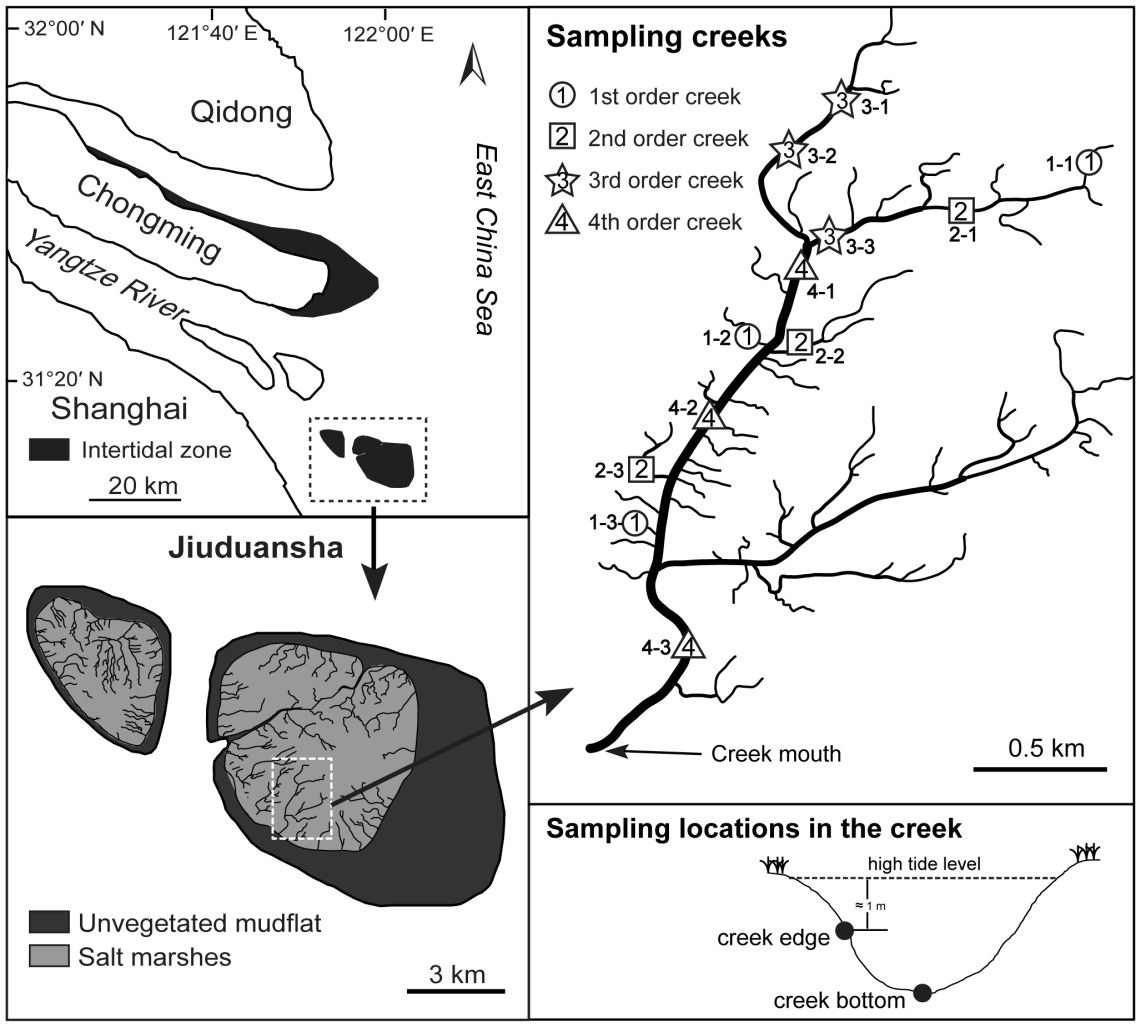
Creeks sampled on the Jiuduansha Island. Samples were collected at two locations (bottom and edge) in each creek.

The study site was located in Shanghai Jiuduansha Wetland National Nature Reserve (31°03′–31°17′N, 121°46′–122°15′E) and covered a total area of 420.2 km^2^ (Figure 1). Jiuduansha, with a history of about only 60 years, is a young intertidal island in the Yangtze River estuary. It was formed mainly due to the connection of flood tidal currents and ebb tidal currents. Since its formation, it has experienced considerable enlargement, heightening and shape modification because of the dynamic tidal fluxes in the estuary. The salt marsh is dominated by the native plants (*Phragmites australis* and *Scirpus mariqueter*) and an exotic plant (*Spartina alterniflora*). The mean annual temperature, precipitation, and salinity is 15.7°C, 1145 mm, and 11.7, respectively. The intertidal zones are flooded by irregular semi-diurnal tide with the tidal range from 2.45 m to 4.96 m (mean tidal range 2.75) [26].

### Sampling design

Monthly samples were collected from a four order creek system during the neap tides between May 2008 to April 2009. We sampled three replicate creeks per stream order (Figure 1). In each creek, we sampled at two locations, the deepest point of the creek (bottom), and an edge position approximately 1 m below the high tide level (edge). All the samples were taken in the intertidal zone and not within the vegetation. At each location, we collected five cores (176.6 cm^2^ in area and 20 cm depth for each core) at intervals of 2–3 m. Sediments from these five cores were mixed to form a composite sample for polychaete collection. Thus, we collected a total of 288 composite samples during the study. The sediment samples were washed through a 0.5 mm mesh sieve. *D. glabra* were sorted and fixed in 10% formalin. The top 5 mm of sediment within a 4×8 cm area was collected at each location and preserved at −20°C for chlorophyll *a* (chl *a*) concentration analysis [27]. Additional sediment samples down to a depth of 10 cm (19.6 cm^2^) were taken for analysis of water content and grain size. Sediment physical parameters (temperature, pH, and conductivity) were directly measured during each month, except May, June, and July, 2008. A global positioning system (GPS) was used to ensure we sampled the same location each month.

### Laboratory procedures

Sediment was dried to constant weight at 80°C to measure water content. Chl *a* content in the sediment was determined using spectrophotometry to represent benthic algae biomass [28]. Algae are an important food source for many primary benthic consumers. Although *D. glabra* do not ingest algae directly, there are indirect relationships between algal biomass and the carnivorous consumer species. Sediment samples for grain size analysis collected from the same location in June, September, and December 2008 and March 2009 were mixed and measured using a LS-POP (VI) laser grain sizer. Geomorphological characters included cross-sectional area, bank slope, bank full elevation, width and depth with the detail measuring methods followed our previous study in [29].

We used this width at setiger 8 (Wd8S) as an indicator of the total length of the polychaetes [30]. The Wd8S of each *D. glabra* individual was measured using a photo imaging system (NIS-Elements D 2.20) to the nearest 0.01 mm. Complete specimens of *D. glabra* were dried at 60°C until constant weight to estimate dry mass (DM). The ash free dry weight (AFDM) was obtained by incinerating at 450°C in a muffle furnace for 8 h.

### Data analysis

We used the relationship between AFDM and Wd8S (AFDM = 0.8082 Wd8S^1.1226^, N = 32, R^2^ = 0.9792) to estimate biomass and secondary production. To estimate secondary production, species were grouped into several size classes at 0.4 mm intervals based on Wd8S.

Secondary production was calculated using the size frequency method [31], [32], expressed as:
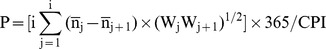
where P is the secondary production (g AFDM m^−2^year^−1^), i is the number of size classes, 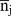 is the mean density in size class j (ind m^−2^), W_j_ is the mean individual weight in size class j (g AFDM), and CPI is the longevity time from hatching to death of the largest size class.

The seasonal VBGF (von Bertalanffy growth function) was used to estimate the longevity of *D. glabra* [33]:
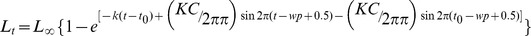
where L_t_ is the length (Wd8S) at time t (mm), L_∞_ is the theoretical maximum length (Wd8S), K is the curvature parameter, C is a constant representing the amplitude of seasonal growth oscillation, t_0_ is the age at zero length (Wd8S), wp is the period when growth is slowest. The value for t_0_ was estimated on the basis of the smallest width found [30]. Life span was calculated based on the age at which 90% of L_∞_ was achieved [17].

Because creeks of the same order have similar width [29], the creek area of each order was calculated based on creek width and its total length. We used ArcGIS 9.3 to measure the length of each creek on Jiuduansha Island based on satellite image data of 2 m resolution.

One-way ANOVA was used to evaluate the effect of creek order on environmental parameters. Two-way or one-way ANOVA was used to evaluate the effects of creek order and sampling location on the density and biomass of *D. glabra*. The effect of creek order on secondary production of *D. glabra* was also evaluated by one-way ANOVA. Post hoc multiple comparisons were performed using the least square differences (LSD) test. Differences were regarded as significant at *P*<0.05. To meet the assumptions of ANOVA, numeric data were log (x+1) transformed before statistical analyses when necessary.

To examine the relationship between secondary production of *D. glabra* and environmental factors, we performed model selection in regression analyses using Akaike's information criterion (AIC) corrected for small sample size (AIC_C_) [34]. Spearman correlation were used to exclude the auto-correlated factors with the absolute coefficients larger than 0.7 [35]. Water content, Chl *a* concentration, conductivity, temperature, pH, clay-silt content, bank full elevation, cross-sectional area and bank slope were considered in models. Creek order was a categorical factor. Numeric data were log transformed before analyses. We calculated the Akaike weight (*w_i_*) and R^2^ for each model. Akaike weight (*w_i_*) represents the probability that the model *i* is the best model among the given set of alternatives [34]. The proportion of variance explained by the model (R^2^) was calculated for each model [36].

## Results

Environmental factors by creeks and orders are showed in Table 1. In creeks across 1st to 4th orders, the ranges of sediment water content, Chl *a* concentration, pH, conductivity, temperature and clay-silt content were 52.60–78.91 (%), 0.02–30.60 (μg/g), 6.39–8.55, 1.52–7.97 (ms/s), 0.55–30.95 (°C) and 65.79–87.31 (%), respectively (Table 1). The highest water content, temperature, pH and sand content were in the 4th order creek. The highest Chl *a* concentration and clay-silt content were in 2nd and 1st order creek, respectively. The values of bank full depth, width, and cross-sectional area increased with increasing creek order.

**Table 1:** Physicochemical parameters and geomorphological characters in creek of each order with one-way ANOVA results showing the effect of creek order.

Environmental factors	Creek order				ANOVA		
	1st	2nd	3rd	4th	*F*	df	*P*
Physicochemical parameters							
Water content (%)	68.55±0.57	68.70±0.72	67.77±0.73	68.79±0.76	0.444	3	0.722
Chl *a* (μg/g)	5.06±0.82^ab^	5.63±1.08^a^	3.52±0.51^b^	0.51±0.20^c^	9.849	3	<0.001
Conductivity (ms/s)	4.30±0.20	4.29±0.22	4.78±0.20	4.59±0.34	0.927	3	0.431
Temperature (°C)	15.49±1.67	15.19±1.69	15.25±1.67	15.64±1.71	0.016	3	0.997
pH	7.59±0.08	7.56±0.08	7.46±0.10	7.73±0.07	1.841	3	0.144
Clay-silt (%)	76.10±1.35^a^	75.17±1.18^a^	75.69±1.08^a^	70.39±0.29^b^	6.315	3	<0.001
Sand (%)	23.90±1.35^a^	24.83±1.18^a^	24.31±1.08^a^	29.61±0.29^b^	6.315	3	<0.001
Geomorphological characters							
Bank full elevation (m)	3.48±0.15	3.66±0. 27	3.51±0.08	3.55±0.13	0.213	3	0.885
Bank full width (m)	12.43±1.39^a^	16.00±1.03^a^	17.93±2.65^a^	29.33±2.57^b^	12.865	3	0.002
Bank full depth (m)	1.82±0.11^a^	2.01±0.16^a^	2.16±0.17^a^	2.57±0.07^b^	6.015	3	0.019
Cross-sectional area (m^2^)	12.69±1.33^a^	17.30±1.97^ab^	22.00±3.44^b^	46.88±5.60^c^	19.042	3	0.001
Bank slope	0.29±0.05	0.26±0.03	0.26±0.05	0.21±0.01	0.850	3	0.505

Shown are Fisher's *F*-ratio (*F*) and *P*-values of ANOVA for testing differences of each variable among creeks of different orders. Different superscript letters (a, b and c) represent a significant difference (*P* < 0.05) among creeks of different orders.

**Figure 2:**
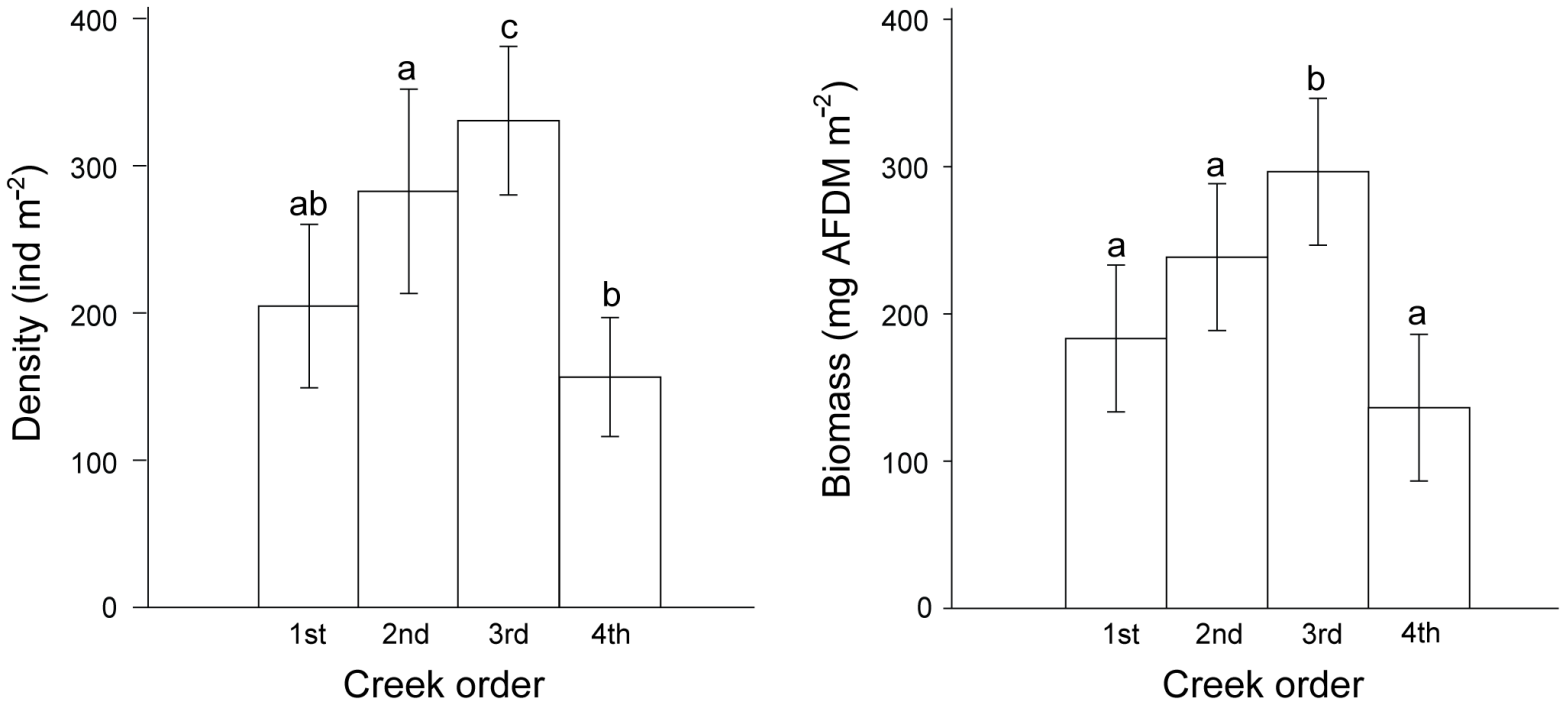
Density and biomass of *Dentinephtys glabra* in creeks of different orders. Error bars represent standard error (n = 72). The same letters above the bars denote non-significant differences and different letters represent a significant difference (*P*<0.05) among creek orders.

**Figure 3:**
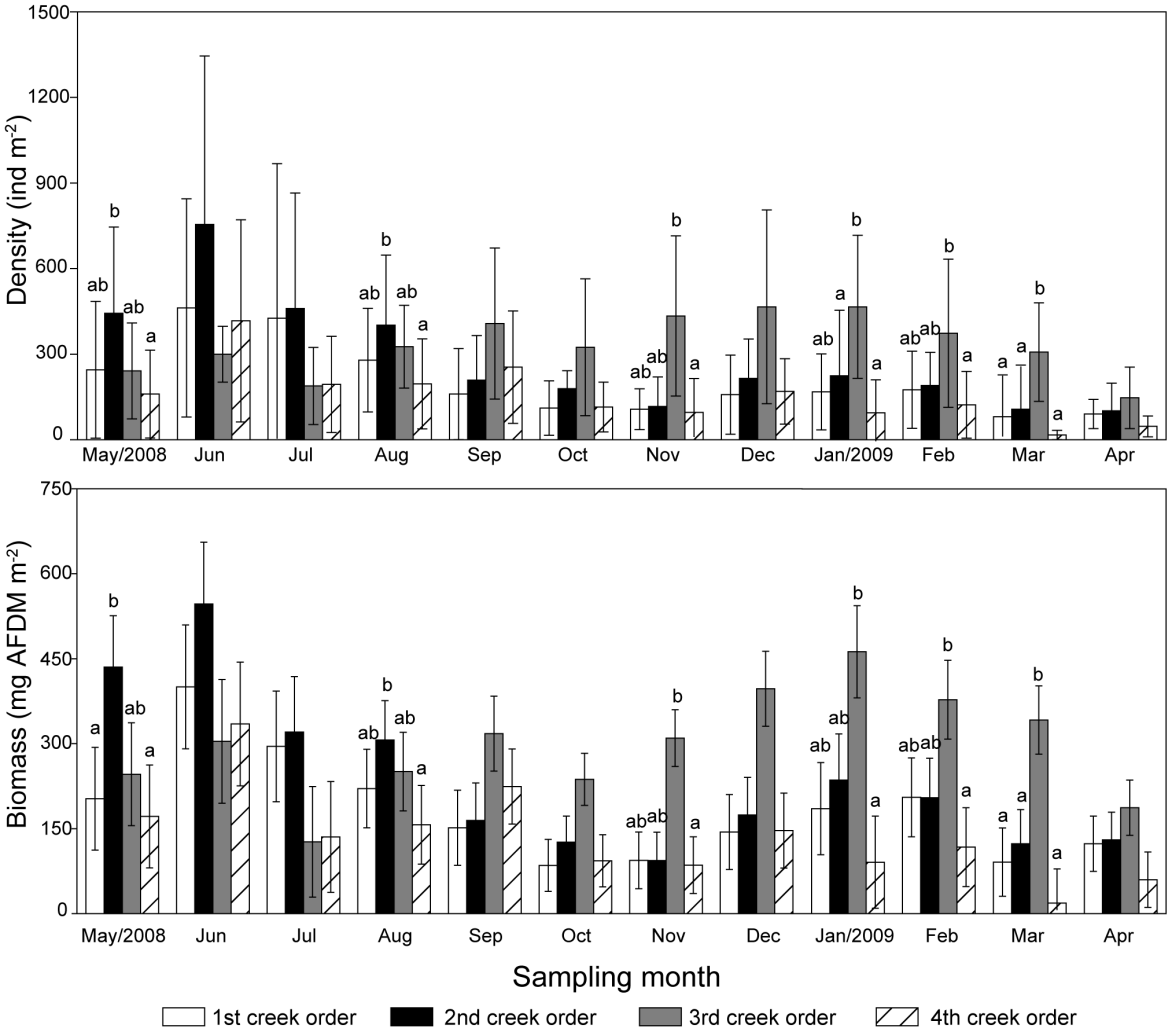
Monthly density and biomass of *Dentinephtys glabra* in creeks of different orders. Error bars represent standard error (n = 6). The same letters above the bars denote non-significant differences and different letters represent a significant difference (*P*<0.05) among creek orders in each month.

**Figure 4:**
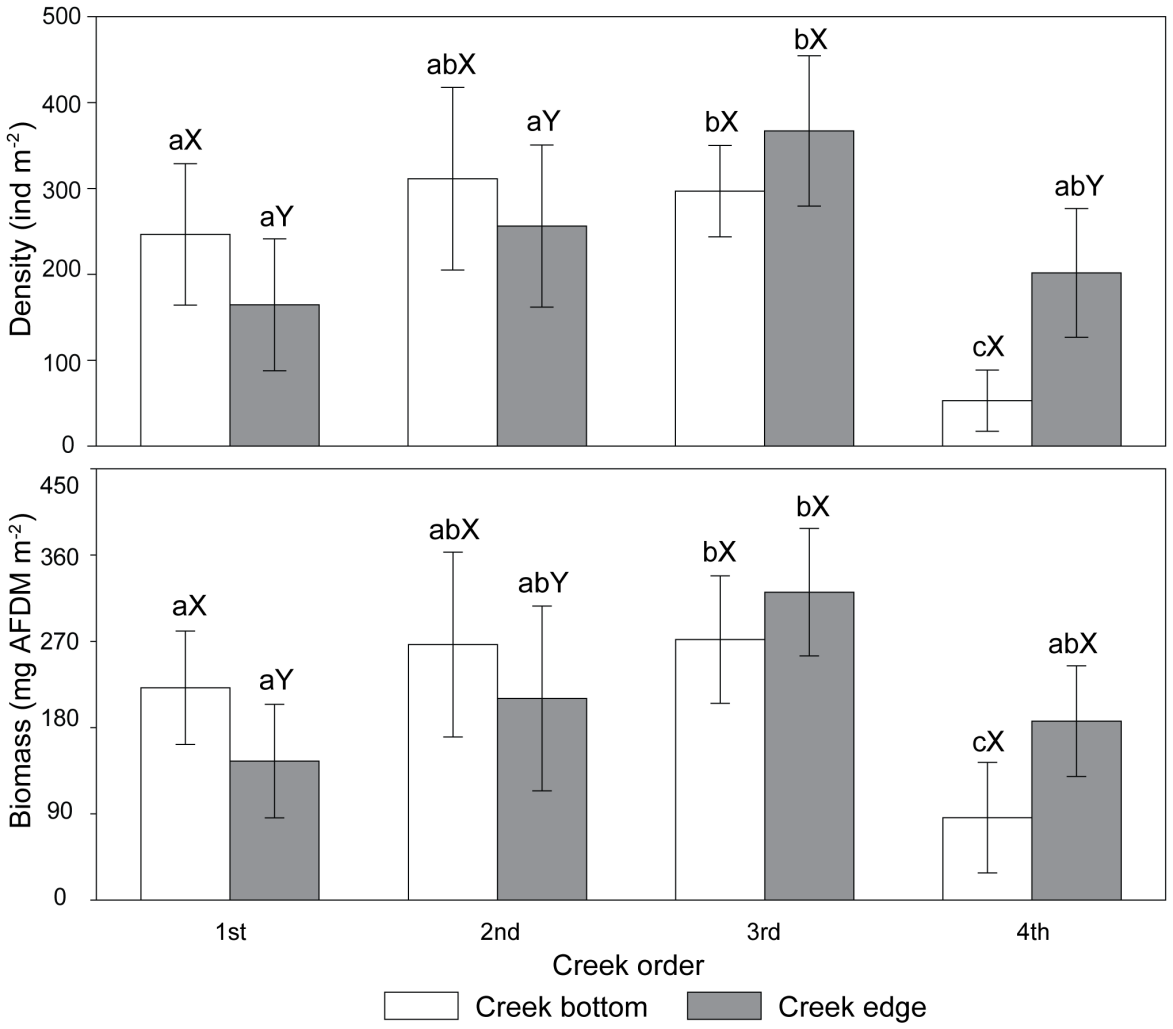
Density, biomass of *Dentinephtys glabra* at bottom and edge sampling locations in creeks of different orders. Error bars represent standard error (n = 36). The same capital letters denote non-significant differences and different capital letters represent a significant difference (*P*<0.05) between bottom and edge locations for creeks of the same order. The same lowercase letters denote non-significant differences and different letters represent a significant difference (*P*<0.05) among creek orders for each sampling location.

The density and biomass of *D. glabra* are shown in Figure 2, 3 and 4. The density and biomass of *D. glabra* was highest in the 3rd order creeks (332.0 ind m^−2^, 296.6 mg AFDM m^−2^), followed by the 2nd order creeks (283.9 ind m^−2^, 238.5 mg AFDM m^−2^) and 1st order creeks (205.6 ind m^−2^, 183.3 mg AFDM m^−2^), and were lowest in the 4th order creeks (157.1 ind m^−2^, 136.3 mg AFDM m^−2^) (Figure 2). The 3rd order creek had significantly higher densities (*P* = 0.0002) and biomass (*P* = 0.0013) of *D. glabra* than other order creeks. This spatial pattern varied with different sampling months. From May to August, the highest density and biomass of *D. glabra* occurred in the 2nd order creeks, whereas between September to April levels were highest in the 3rd order creeks (Figure 3).

For the 1st and 2nd order creeks, the density (1st: *P* = 0.0222; 2nd: *P* = 0.0160) and biomass (1st: *P* = 0.0331; 2nd: *P* = 0.0118) of *D. glabra* were significantly higher at the bottom locations than at the edge locations (Figure 4). However, the trend was reversed in the 3rd and 4th order creeks. The density and biomass of *D. glabra* were highest at the edge location of the 3rd order creeks (367.1 ind m^−2^, 321.3 mg AFDM m^−2^), and lowest at the bottom location of the 4th order creeks (88.7 ind m^−2^, 85.9 mg AFDM m^−2^).

The secondary production of *D. glabra* is shown in Table 2. A total of 6222 individuals were collected during the year-round sampling with the mean Wd8S ranging from 0.84 to 1.59 mm. Using the size frequency method (Table S1–S4), we estimated secondary production to be 286.0, 559.7, 448.2, and 247.4 mg AFDM m^−2^year^−1^ for 1st to 4th order creeks, with annual P/B ratios of 1.56, 2.39, 1.69, and 1.75, respectively (Table 2). Secondary production differed among creeks of different orders. The secondary production in the 2nd order creeks was 2.26 times greater than in the 4th order creeks.

**Table 2:** Estimation of the total *Dentinephtys glabra* production in Jiuduansha creeks on an order by order basis with one-way ANOVA results showing the effect of creek order.

Creek order	*Dentinephtys glabra*				Total creeks of Jiuduansha			Production of *D. glabra* in total creeks of Jiuduansha (10^6^ mg AFDM year^−1^)
	K (year^−1^)	Life span (years)	Production (mg AFDM m^−2^year^−1^)	P/B	Total length (10^3^m)	Average width (m)	Total area (10^3^ m^2^)	
1st	1.05±0.04	2.39±0.10	286.0±83.6^ab^	1.56±0.05	121.5	12.4	1506.9	431.0
2nd	1.37±0.37	2.08±0.41	559.7±90.1^a^	2.39±0.43	54.7	16.0	875.1	489.8
3rd	1.23±0.29	2.22±0.39	448.2±45.1^ab^	1.69±0.35	22.9	17.9	409.1	183.3
4th	1.07±0.07	2.36±0.12	247.4±76.4^b^	1.75±0.18	5.5	29.3	160.0	39.6
Total							2952.0	1143.8

Different superscript letters (a and b) represent a significant difference (*P*<0.05) among creeks of different orders.

The secondary production of creek system in Jiuduansha marsh is shown in table 2. The creek system in Jiuduansha marsh was 2952.0×10^3^ m^2^ in area, with the 1st order creeks accounting for 51.1% of the total area (1507.9×10^3^ m^2^). The average and total production of *D. glabra* in creeks of the Jiuduansha marsh was estimated to be 387.2 mg AFDM m^−2^year^−1^ and 1143.8×10^6^ mg AFDM year^−1^.

The top five selected AIC models for the relationship between secondary production of *D. glabra* and environmental factors are shown in Table 3. During the analyses, the cross-sectional area was selected as an integrated performance for bank full depth and width. Clay-silt content was selected to represent grain size of the sediment. All five models had the similar *w_i_* value, and included cross-sectional area, bank slope and Chl *a* concentration, suggesting the importance of these variables.

**Table 3:** Results of model selection using Akaike's information criterion (AIC) for the relationship between secondary production of *Dentinephtys glabra* and environmental factors.

Model	AICc	△AICc	*w_i_*	R^2^	Z values									
					Intercept	Chl *a*	Clay	Cond	Cross	Elevation	pH	Slope	Temp	Water
1	219.49	0	0.07	0.65	0.86	−0.16			−0.47		0.05	−0.24		
2	219.70	0.21	0.06	0.65	0.86	−0.17		0.04	−0.49			−0.24		
3	219.86	0.37	0.06	0.65	0.86	−0.16			−0.46			−0.24	−0.03	
4	220.72	1.22	0.04	0.65	0.85	−0.14	−0.07		−0.49		0.04	−0.26		
5	220.98	1.49	0.03	0.65	0.85	−0.15	−0.07	0.03	−0.50			−0.26		

Only the top five models with Akaike weight (*w_i_*) and R^2^ values are shown. Regression coefficients were expressed as Z values (estimate/SE).

Factors are abbreviated as Cond (conductivity), Cross (cross-sectional area), Slope (bank slope), Water (Water content), Chl *a* (Chl *a* concentration), Temp (temperature), Clay (clay-silt content).

## Discussion

### Spatial distribution

Our results showed that the highest density and biomass of *D. glabra* occurred in intermediate order creeks. The density of the polychaete *Streblospio benedicti* has been reported to increase with increasing creek order because the creeks of higher order had more constant physical characteristics [37]. Interestingly, the inverse trend of higher polychaete densities in lower order creeks than in higher order creeks has also been documented [11], [24], [38]. Taken together, these observations suggest that the macrofaunal utilization pattern of creeks along an order gradient differs with species or regions.

Researchers have found that the density of polychaetes was higher in the edge sites than in the bottom sites [23]. Interestingly, the densities and biomass of *D. glabra* were higher in edge locations than in bottom locations in the high order (3rd and 4th order) creeks, and lowest in the edge locations of low order (1st and 2nd order) creeks. Essential abiotic properties including sediment grain size and tidal forces may influence the macrobenthos distributions [39]. For example, the density of polychaete *Capitella* spp. was positively correlated with percent silt [40]. In high order creeks, macrobenthos may be exposed to more intense hydrodynamic forces in the bottom sites than in edge sites, resulting in lower abundance at bottom sites [23].

The pattern of polychaete distribution may also be influenced by other organisms. For example, the presence of burrowing crabs (*Chasmagnathus granulatus*) reduces the abundance of polychaetes [41]. This is because the crabs reduce the burrowing depth of polychaetes, thereby increasing the availability of the polychaetes to shorebirds [41]. In salt marsh creeks of the Yangtze River estuary, the abundance of the crab *Ilyoplax deschampsi* was higher in edge sites than in bottom sites [42]. The effects of *I. deschampsi* on *D. glabra* may be more evident in lower order creeks where *I. deschampsi* is more abundant than in higher order creeks [24]. This may also explain the difference in *D. glabra* abundance between edge and bottom sites between the higher and lower order creeks. Predator is also an important factor to influence the secondary production of polychaete [43]. The secondary production of *D. glabra* was at least two fold higher in intermediate order creeks than in both low and high order creeks. In high order creeks, longer tidal inundation exposed *D. glabra* to carnivorous fishes for longer time than in low order creeks. However, in the low order creeks, benthic polychaete *D. glabra* would be more exposed to shorebirds because of limited tidal inundation [44]. The pattern that higher secondary production of *D. glabra* occurred in intermediate order creeks may reflect the predatory stress to some extent.

### Secondary production

Studies evaluating the secondary production of Nephtyidae are very rare, in fact only three species (*Nephtys hombergii*, *N. australiensis* and *N. picta*) have been studied to date. The secondary production of *N. australiensis* was 48.0 mg AFDW m^−2^year^−1^ [45]. The secondary production of *N. hombergii* ranged from 92.0 [46] to 7335.0 mg AFDW m^−2^year^−1^ [47], and that of *N. picta* from 95.0 to 628.0 mg AFDW m^−2^year^−1^ [48]. Compared with these reports, secondary production obtained for *D. glabra* in this study was within the range of 48.0 to 7335.0 mg AFDM m^−2^year^−1^.

Tidal influences result in wide fluctuations of environmental variables within intertidal creek networks [37]. Turbidity and water temperature decreased, while pH, conductivity, flow, and drainage basin area increased as creek order increased [14]. In our study, Chl *a* concentration and sediment grain size were found to be different among different creek orders. Secondary production is influenced by many physicochemical factors, e.g. temperature, dissolved oxygen, substrate [49]. Secondary production of the polychaete *N. hombergii* differed among areas of different organic matter content [44]. Research found the decrease of secondary production of polychaete *Nereis diversicolor* may be attributed to the increase of salinity and the decrease of organic matter content in the estuary of Oued Souss [50]. In the top fitting AIC models, secondary production of *D. glabra* was mainly associated with geomorphological characters including cross-sectional area and bank slope. This may reflect that the hydrodynamic forces are essential factors for secondary production of macrobenthos in salt marshes. Animals are not evenly distributed across wetland landscapes and the distributions of secondary production change with the high complex landscape topography [51]. Recently, researchers have incorporated the use of GIS analysis to estimate the secondary production for a region. For example, they calculated the area of each sub-habitat (different distances from the marsh edge) using GIS and then calculated the total secondary production of crustaceans for the Galveston Bay salt marsh [52]. In the present study, because of the difference in *D. glabra* production among different order creeks, we think it is necessary to combine area estimation using GIS analysis to estimate total secondary production in the Jiuduansha marsh. Based on this approach, the total production of *D. glabra* in Jiuduansha was 1143.8×10^6^ mg AFDM year^−1^. We believe that improved knowledge of the spatial heterogeneity in secondary production combined with landscape ecology is critical to quantify the material cycling and energy flow for the creek networks.

## Conclusions

The major findings of this study were: (1) the density and biomass of the polychaete *D. glabra* differed significantly among creeks of different orders, but was highest in intermediate order creeks. The highest density and biomass were found in the 3rd order creeks and the lowest were in the 4th order creeks. (2) In lower order creeks, its density and biomass were higher in the creek bottom than in creek edge sites. However, the reverse was true in higher order creeks. (3) The secondary production of *D. glabra* was much higher in intermediate order creeks than in low and high order creeks. Its secondary production in the 2nd order creeks (559.7 mg AFDM m^−2^ year^−1^) was 2.26 times greater than in 4th order creeks.

Polychaetes are a dominant group and key linkage in the food web of salt marsh ecosystems. The research about polychaete secondary production can be an indicator to evaluate the health of the ecosystems. Our findings indicated the importance of creek order in influencing the abundance and secondary production of macrobenthic animals such as polychaetes. This suggests that future studies of infauna on tidal creek systems should carefully consider the within-system differences. The spatial variability in prey production can, in turn, influence the habitat utilization by predators including nektonic fish and crustaceans, and birds. Thus, the value of different microhabitats should be considered in assessing marsh functions and implementing management and restoration strategies for the purposes of biodiversity conservation.

## Supporting Information

Table S1 **Annual production of** ***Dentinephtys glabra*** **at 1st order creeks estimated by the size-frequency method.**
(DOC)

Table S2 **Annual production of** ***Dentinephtys glabra*** **at 2nd order creeks estimated by the size-frequency method.**
(DOC)

Table S3 **Annual production of** ***Dentinephtys glabra*** **at 3rd order creeks estimated by the size-frequency method.**
(DOC)

Table S4 **Annual production of** ***Dentinephtys glabra*** **at 4th order creeks estimated by the size-frequency method.**
(DOC)
