# Supplementary material for: Variability of Polychaete Secondary Production in Intertidal Creek Networks along a Stream-Order Gradient
Source: PLoS One. 2014 May 9;9(5):e97287. doi: 10.1371/journal.pone.0097287 (PMC4016305; doi:10.1371/journal.pone.0097287)
Supplement: Table S1 — Annual production of Dentinephtys glabra at 1st order creeks estimated by the size-frequency method. (DOC) [file pone.0097287.s001.doc]

**Table S1.** Annual production of *Dentinephtys glabra* at 1st order creeks estimated by the size-frequency method.

| Creek number | Size group | Density | No loss | Biomass | Mean wt | Mean wt at loss | Wt loss | Production |
| --- | --- | --- | --- | --- | --- | --- | --- | --- |
|  | (mm) | (ind/m^2^) | (ind/m^2^) | (mg AFDM/m^2^) | (mg AFDM) | (mg AFDM) | (mg AFDM/m^2^) | (mg AFDM/m^2^) |
| 1-1 | 0.05-0.45 | 0.472 | -138.240 | 0.151 | 0.320 | 0.433 | -59.913 | -599.133 |
|  | 0.45-0.85 | 138.712 | -15.570 | 81.360 | 0.587 | 0.679 | -10.570 | -105.696 |
|  | 0.85-1.25 | 154.282 | 130.691 | 121.219 | 0.786 | 0.974 | 127.263 | 1272.632 |
|  | 1.25-1.65 | 23.590 | 5.190 | 28.470 | 1.207 | 1.388 | 7.203 | 72.029 |
|  | 1.65-2.05 | 18.401 | 10.380 | 29.368 | 1.596 | 1.751 | 18.172 | 181.717 |
|  | 2.05-2.45 | 8.021 | 6.605 | 15.402 | 1.920 | 2.188 | 14.455 | 144.546 |
|  | 2.45-2.85 | 1.415 | 0.000 | 3.530 | 2.494 | 2.641 | 0.000 | 0.000 |
|  | 2.85-3.25 | 1.415 | 1.415 | 3.960 | 2.798 | 3.009 | 4.259 | 42.590 |
|  | 3.25-3.65 | 0.000 | -0.472 | 0.000 | 3.236 | 3.448 | -1.627 | -16.268 |
|  | 3.65-4.05 | 0.472 | 0.472 | 1.733 | 3.674 | 3.674 | 1.733 | 17.334 |
| 1-2 | 0.05-0.45 | 0.472 | -28.780 | 0.027 | 0.057 | 0.187 | -5.382 | -53.824 |
|  | 0.45-0.85 | 29.252 | -47.181 | 17.889 | 0.612 | 0.714 | -33.670 | -336.697 |
|  | 0.85-1.25 | 76.433 | 49.540 | 63.650 | 0.833 | 0.992 | 49.137 | 491.373 |
|  | 1.25-1.65 | 26.893 | 12.267 | 31.771 | 1.181 | 1.377 | 16.889 | 168.892 |
|  | 1.65-2.05 | 14.626 | 10.380 | 23.468 | 1.605 | 1.770 | 18.370 | 183.696 |
|  | 2.05-2.45 | 4.246 | 3.774 | 8.289 | 1.952 | 2.192 | 8.275 | 82.754 |
|  | 2.45-2.85 | 0.472 | 0.472 | 1.162 | 2.463 | 2.463 | 1.162 | 11.619 |
|  | 2.85-3.25 | 0.000 | 0.000 | 0.000 | 0.000 | 0.000 | 0.000 | 0.000 |
|  | 3.25-3.65 | 0.000 | 0.000 | 0.000 | 0.000 | 0.000 | 0.000 | 0.000 |
|  | 3.65-4.05 | 0.000 | 0.000 | 0.000 | 0.000 | 0.000 | 0.000 | 0.000 |
| 1-3 | 0.05-0.45 | 0.000 | 0.000 | 0.000 | 0.000 | 0.000 | 0.000 | 0.000 |
|  | 0.45-0.85 | 17.457 | -36.329 | 10.091 | 0.578 | 0.708 | -25.710 | -257.101 |
|  | 0.85-1.25 | 53.786 | 23.119 | 46.602 | 0.866 | 1.013 | 23.409 | 234.094 |
|  | 1.25-1.65 | 30.668 | 17.457 | 36.291 | 1.183 | 1.359 | 23.717 | 237.168 |
|  | 1.65-2.05 | 13.211 | 10.852 | 20.605 | 1.560 | 1.781 | 19.325 | 193.251 |
|  | 2.05-2.45 | 2.359 | 2.359 | 4.797 | 2.033 | 2.033 | 4.797 | 47.967 |
|  | 2.45-2.85 | 0.000 | 0.000 | 0.000 | 0.000 | 0.000 | 0.000 | 0.000 |
|  | 2.85-3.25 | 0.000 | 0.000 | 0.000 | 0.000 | 0.000 | 0.000 | 0.000 |
|  | 3.25-3.65 | 0.000 | 0.000 | 0.000 | 0.000 | 0.000 | 0.000 | 0.000 |
|  | 3.65-4.05 | 0.000 | 0.000 | 0.000 | 0.000 | 0.000 | 0.000 | 0.000 |
