# Supplementary material for: Variability of Polychaete Secondary Production in Intertidal Creek Networks along a Stream-Order Gradient
Source: PLoS One. 2014 May 9;9(5):e97287. doi: 10.1371/journal.pone.0097287 (PMC4016305; doi:10.1371/journal.pone.0097287)
Supplement: Table S2 — Annual production of Dentinephtys glabra at 2nd order creeks estimated by the size-frequency method. (DOC) [file pone.0097287.s002.doc]

**Table S2.** Annual production of *Dentinephtys glabra* at 2nd order creeks estimated by the size-frequency method.

| Creek number | Size group | Density | No loss | Biomass | Mean wt | Mean wt at loss | Wt loss | Production |
| --- | --- | --- | --- | --- | --- | --- | --- | --- |
|  | (mm) | (ind/m^2^) | (ind/m^2^) | (mg AFDM/m^2^) | (mg AFDM) | (mg AFDM) | (mg AFDM/m^2^) | (mg AFDM/m^2^) |
| 2-1 | 0.05-0.45 | 0.000 | 0.000 | 0.000 | 0.000 | 0.000 | 0.000 | 0.000 |
|  | 0.45-0.85 | 134.466 | -20.760 | 78.032 | 0.580 | 0.677 | -14.047 | -140.471 |
|  | 0.85-1.25 | 155.225 | 129.748 | 122.471 | 0.789 | 0.982 | 127.422 | 1274.222 |
|  | 1.25-1.65 | 25.478 | 0.000 | 31.145 | 1.222 | 1.395 | 0.000 | 0.000 |
|  | 1.65-2.05 | 25.478 | 11.795 | 40.572 | 1.592 | 1.777 | 20.955 | 209.546 |
|  | 2.05-2.45 | 13.682 | 12.739 | 27.117 | 1.982 | 2.204 | 28.071 | 280.713 |
|  | 2.45-2.85 | 0.944 | 0.944 | 2.312 | 2.450 | 2.450 | 2.312 | 23.119 |
|  | 2.85-3.25 | 0.000 | 0.000 | 0.000 | 0.000 | 0.000 | 0.000 | 0.000 |
|  | 3.25-3.65 | 0.000 | 0.000 | 0.000 | 0.000 | 0.000 | 0.000 | 0.000 |
|  | 3.65-4.05 | 0.000 | 0.000 | 0.000 | 0.000 | 0.000 | 0.000 | 0.000 |
| 2-2 | 0.05-0.45 | 0.472 | -132.578 | 0.126 | 0.268 | 0.387 | -51.339 | -513.392 |
|  | 0.45-0.85 | 133.050 | 36.801 | 74.419 | 0.559 | 0.659 | 24.268 | 242.676 |
|  | 0.85-1.25 | 96.249 | 79.264 | 74.827 | 0.777 | 0.976 | 77.348 | 773.476 |
|  | 1.25-1.65 | 16.985 | 5.662 | 20.804 | 1.225 | 1.408 | 7.970 | 79.699 |
|  | 1.65-2.05 | 11.323 | 8.021 | 18.319 | 1.618 | 1.784 | 14.307 | 143.066 |
|  | 2.05-2.45 | 3.303 | 2.831 | 6.495 | 1.967 | 2.158 | 6.108 | 61.079 |
|  | 2.45-2.85 | 0.472 | 0.000 | 1.117 | 2.367 | 2.572 | 0.000 | 0.000 |
|  | 2.85-3.25 | 0.472 | 0.472 | 1.318 | 2.793 | 2.793 | 1.318 | 13.180 |
|  | 3.25-3.65 | 0.000 | 0.000 | 0.000 | 0.000 | 0.000 | 0.000 | 0.000 |
|  | 3.65-4.05 | 0.000 | 0.000 | 0.000 | 0.000 | 0.000 | 0.000 | 0.000 |
| 2-3 | 0.05-0.45 | 0.000 | 0.000 | 0.000 | 0.000 | 0.000 | 0.000 | 0.000 |
|  | 0.45-0.85 | 52.371 | -58.033 | 30.341 | 0.579 | 0.705 | -40.908 | -409.077 |
|  | 0.85-1.25 | 110.403 | 54.730 | 94.691 | 0.858 | 1.009 | 55.198 | 551.980 |
|  | 1.25-1.65 | 55.674 | 43.878 | 66.027 | 1.186 | 1.352 | 59.318 | 593.182 |
|  | 1.65-2.05 | 11.795 | 8.021 | 18.177 | 1.541 | 1.722 | 13.813 | 138.131 |
|  | 2.05-2.45 | 3.774 | 3.774 | 7.264 | 1.925 | 1.925 | 7.264 | 72.644 |
|  | 2.45-2.85 | 0.000 | 0.000 | 0.000 | 0.000 | 0.000 | 0.000 | 0.000 |
|  | 2.85-3.25 | 0.000 | 0.000 | 0.000 | 0.000 | 0.000 | 0.000 | 0.000 |
|  | 3.25-3.65 | 0.000 | 0.000 | 0.000 | 0.000 | 0.000 | 0.000 | 0.000 |
|  | 3.65-4.05 | 0.000 | 0.000 | 0.000 | 0.000 | 0.000 | 0.000 | 0.000 |
