# Supplementary material for: Variability of Polychaete Secondary Production in Intertidal Creek Networks along a Stream-Order Gradient
Source: PLoS One. 2014 May 9;9(5):e97287. doi: 10.1371/journal.pone.0097287 (PMC4016305; doi:10.1371/journal.pone.0097287)
Supplement: Table S3 — Annual production of Dentinephtys glabra at 3rd order creeks estimated by the size-frequency method. (DOC) [file pone.0097287.s003.doc]

**Table S3.** Annual production of *Dentinephtys glabra* at 3rd order creeks estimated by the size-frequency method.

| Creek number | Size group | Density | No loss | Biomass | Mean wt | Mean wt at loss | Wt loss | Production |
| --- | --- | --- | --- | --- | --- | --- | --- | --- |
|  | (mm) | (ind/m^2^) | (ind/m^2^) | (mg AFDM/m^2^) | (mg AFDM) | (mg AFDM) | (mg AFDM/m^2^) | (mg AFDM/m^2^) |
| 3-1 | 0.05-0.45 | 0.944 | -47.181 | 0.299 | 0.317 | 0.429 | -20.247 | -202.474 |
|  | 0.45-0.85 | 48.125 | -27.837 | 27.935 | 0.580 | 0.690 | -19.217 | -192.172 |
|  | 0.85-1.25 | 75.961 | 50.955 | 62.367 | 0.821 | 0.996 | 50.769 | 507.694 |
|  | 1.25-1.65 | 25.006 | 14.626 | 30.235 | 1.209 | 1.398 | 20.441 | 204.411 |
|  | 1.65-2.05 | 10.380 | 7.549 | 16.768 | 1.615 | 1.773 | 13.386 | 133.861 |
|  | 2.05-2.45 | 2.831 | 0.944 | 5.510 | 1.946 | 2.133 | 2.013 | 20.127 |
|  | 2.45-2.85 | 1.887 | 0.944 | 4.411 | 2.337 | 2.577 | 2.431 | 24.315 |
|  | 2.85-3.25 | 0.944 | 0.944 | 2.681 | 2.841 | 2.841 | 2.681 | 26.806 |
|  | 3.25-3.65 | 0.000 | 0.000 | 0.000 | 0.000 | 0.000 | 0.000 | 0.000 |
|  | 3.65-4.05 | 0.000 | 0.000 | 0.000 | 0.000 | 0.000 | 0.000 | 0.000 |
| 3-2 | 0.05-0.45 | 0.472 | -81.151 | 0.141 | 0.298 | 0.415 | -33.667 | -336.674 |
|  | 0.45-0.85 | 81.623 | -148.620 | 47.165 | 0.578 | 0.691 | -102.737 | -1027.369 |
|  | 0.85-1.25 | 230.243 | 173.154 | 190.403 | 0.827 | 0.984 | 170.365 | 1703.652 |
|  | 1.25-1.65 | 57.089 | 40.104 | 66.828 | 1.171 | 1.369 | 54.906 | 549.062 |
|  | 1.65-2.05 | 16.985 | 12.267 | 27.198 | 1.601 | 1.785 | 21.893 | 218.934 |
|  | 2.05-2.45 | 4.718 | 1.415 | 9.385 | 1.989 | 2.182 | 3.089 | 30.891 |
|  | 2.45-2.85 | 3.303 | 3.303 | 7.908 | 2.395 | 2.395 | 7.908 | 79.083 |
|  | 2.85-3.25 | 0.000 | 0.000 | 0.000 | 0.000 | 0.000 | 0.000 | 0.000 |
|  | 3.25-3.65 | 0.000 | 0.000 | 0.000 | 0.000 | 0.000 | 0.000 | 0.000 |
|  | 3.65-4.05 | 0.000 | 0.000 | 0.000 | 0.000 | 0.000 | 0.000 | 0.000 |
| 3-3 | 0.05-0.45 | 0.472 | -87.757 | 0.147 | 0.311 | 0.426 | -37.362 | -373.624 |
|  | 0.45-0.85 | 88.228 | -152.394 | 51.397 | 0.583 | 0.692 | -105.483 | -1054.835 |
|  | 0.85-1.25 | 240.623 | 163.246 | 197.896 | 0.822 | 0.980 | 159.995 | 1599.954 |
|  | 1.25-1.65 | 77.377 | 58.033 | 90.373 | 1.168 | 1.376 | 79.877 | 798.769 |
|  | 1.65-2.05 | 19.344 | 11.323 | 31.378 | 1.622 | 1.767 | 20.014 | 200.140 |
|  | 2.05-2.45 | 8.021 | 7.549 | 15.447 | 1.926 | 2.085 | 15.740 | 157.405 |
|  | 2.45-2.85 | 0.472 | -0.472 | 1.065 | 2.257 | 2.554 | -1.205 | -12.052 |
|  | 2.85-3.25 | 0.944 | 0.944 | 2.727 | 2.890 | 2.890 | 2.727 | 27.275 |
|  | 3.25-3.65 | 0.000 | 0.000 | 0.000 | 0.000 | 0.000 | 0.000 | 0.000 |
|  | 3.65-4.05 | 0.000 | 0.000 | 0.000 | 0.000 | 0.000 | 0.000 | 0.000 |
