# Supplementary material for: Variability of Polychaete Secondary Production in Intertidal Creek Networks along a Stream-Order Gradient
Source: PLoS One. 2014 May 9;9(5):e97287. doi: 10.1371/journal.pone.0097287 (PMC4016305; doi:10.1371/journal.pone.0097287)
Supplement: Table S4 — Annual production of Dentinephtys glabra at 4th order creeks estimated by the size-frequency method. (DOC) [file pone.0097287.s004.doc]

**Table S4.** Annual production of *Dentinephtys glabra* at 4th order creeks estimated by the size-frequency method.

| Creek number | Size group | Density | No loss | Biomass | Mean wt | Mean wt at loss | Wt loss | Production |
| --- | --- | --- | --- | --- | --- | --- | --- | --- |
|  | (mm) | (ind/m^2^) | (ind/m^2^) | (mg AFDM/m^2^) | (mg AFDM) | (mg AFDM) | (mg AFDM/m^2^) | (mg AFDM/m^2^) |
| 4-1 | 0.05-0.45 | 0.472 | -29.252 | 0.152 | 0.321 | 0.418 | -12.233 | -122.328 |
|  | 0.45-0.85 | 29.724 | -6.134 | 16.178 | 0.544 | 0.670 | -4.107 | -41.069 |
|  | 0.85-1.25 | 35.858 | 30.196 | 29.537 | 0.824 | 0.966 | 29.155 | 291.552 |
|  | 1.25-1.65 | 5.662 | 0.000 | 6.408 | 1.132 | 1.354 | 0.000 | 0.000 |
|  | 1.65-2.05 | 5.662 | 4.246 | 9.175 | 1.621 | 1.828 | 7.761 | 77.607 |
|  | 2.05-2.45 | 1.415 | 0.944 | 2.917 | 2.061 | 2.155 | 2.034 | 20.337 |
|  | 2.45-2.85 | 0.472 | 0.472 | 1.063 | 2.254 | 2.254 | 1.063 | 10.632 |
|  | 2.85-3.25 | 0.000 | 0.000 | 0.000 | 0.000 | 0.000 | 0.000 | 0.000 |
|  | 3.25-3.65 | 0.000 | 0.000 | 0.000 | 0.000 | 0.000 | 0.000 | 0.000 |
|  | 3.65-4.05 | 0.000 | 0.000 | 0.000 | 0.000 | 0.000 | 0.000 | 0.000 |
| 4-2 | 0.05-0.45 | 0.000 | 0.000 | 0.000 | 0.000 | 0.000 | 0.000 | 0.000 |
|  | 0.45-0.85 | 57.089 | -25.950 | 33.303 | 0.583 | 0.682 | -17.705 | -177.052 |
|  | 0.85-1.25 | 83.038 | 66.997 | 66.266 | 0.798 | 0.988 | 66.192 | 661.924 |
|  | 1.25-1.65 | 16.042 | 5.190 | 19.622 | 1.223 | 1.408 | 7.308 | 73.079 |
|  | 1.65-2.05 | 10.852 | 5.190 | 17.590 | 1.621 | 1.805 | 9.365 | 93.654 |
|  | 2.05-2.45 | 5.662 | 3.774 | 11.374 | 2.009 | 2.201 | 8.306 | 83.065 |
|  | 2.45-2.85 | 1.887 | 1.415 | 4.550 | 2.411 | 2.631 | 3.724 | 37.242 |
|  | 2.85-3.25 | 0.472 | 0.472 | 1.355 | 2.872 | 2.872 | 1.355 | 13.549 |
|  | 3.25-3.65 | 0.000 | 0.000 | 0.000 | 0.000 | 0.000 | 0.000 | 0.000 |
|  | 3.65-4.05 | 0.000 | 0.000 | 0.000 | 0.000 | 0.000 | 0.000 | 0.000 |
| 4-3 | 0.05-0.45 | 0.944 | -61.335 | 0.225 | 0.239 | 0.374 | -22.964 | -229.637 |
|  | 0.45-0.85 | 62.279 | -50.484 | 36.581 | 0.587 | 0.685 | -34.587 | -345.873 |
|  | 0.85-1.25 | 112.762 | 91.059 | 90.111 | 0.799 | 0.982 | 89.431 | 894.315 |
|  | 1.25-1.65 | 21.703 | 10.852 | 26.197 | 1.207 | 1.377 | 14.938 | 149.379 |
|  | 1.65-2.05 | 10.852 | 5.662 | 17.036 | 1.570 | 1.768 | 10.008 | 100.081 |
|  | 2.05-2.45 | 5.190 | 3.303 | 10.330 | 1.990 | 2.239 | 7.393 | 73.934 |
|  | 2.45-2.85 | 1.887 | 0.944 | 4.752 | 2.518 | 2.649 | 2.499 | 24.994 |
|  | 2.85-3.25 | 0.944 | 0.472 | 2.629 | 2.787 | 2.964 | 1.398 | 13.984 |
|  | 3.25-3.65 | 0.472 | 0.472 | 1.487 | 3.152 | 3.152 | 1.487 | 14.874 |
|  | 3.65-4.05 | 0.000 | 0.000 | 0.000 | 0.000 | 0.000 | 0.000 | 0.000 |
